# Uma Causa Rara e Tratamento da Fibrilação Ventricular: Toxicidade do 5-Fluorouracil

**DOI:** 10.36660/abc.20230217

**Published:** 2023-12-19

**Authors:** Kutluhan Eren Hazir, Cenk Sari

**Affiliations:** 1 Izmir Provincial Health Directorate Izmir University of Health Sciences Tepecik Training and Research Hospital Ringgold standard institution Department of Cardiology Konak İzmir Turquia Izmir Provincial Health Directorate Izmir University of Health Sciences Tepecik Training and Research Hospital Ringgold standard institution - Department of Cardiology, Konak, İzmir – Turquia

**Keywords:** Fluoruracila, Fibrilação Ventricular, Oncologia

## Abstract

Um homem de 65 anos com histórico de carcinoma de língua procurou o pronto-socorro com contrações insensíveis estando em casa. Ele estava em terapia com 5-fluorouracil (5-FU) na época. O paciente foi desfibrilado e intubado porque a fibrilação ventricular (FV) se desenvolveu durante o monitoramento no pronto-socorro. A ecocardiografia mostrou que a fração de ejeção do ventrículo esquerdo (FEVE) era de 70% e a espessura do septo interventricular era de 15 mm. A angiografia coronária não revelou qualquer estenose crítica. A ressonância magnética cardíaca (RMC) não mostrou anormalidade de perfusão, fibrose ou cicatriz sugestiva de envolvimento cardíaco. Foi sugerido que a arritmia do paciente estava relacionada principalmente à cardiotoxicidade induzida pelo 5-FU.

O fato de as causas secundárias terem sido proeminentes em nosso caso, de nenhuma patologia cardíaca óbvia que pudesse causar arritmia ter sido encontrada no exame detalhado e de a arritmia não ter recorrido durante a internação hospitalar, que durou até 15 dias, nos levou a acreditar que esse paciente poderia receber alta sem um cardioversor-desfibrilador implantável. Nosso caso foi apresentado para contribuir com a literatura.

## Introdução

O 5-fluorouracil (5-FU) é um antimetabólito da pirimidina usado no tratamento de tumores sólidos.^1^ O 5-FU pode causar vários efeitos colaterais cardiovasculares com uma frequência de 1,2-18%.^1^, ^2^ Entre esses efeitos colaterais, são comuns a angina e o infarto do miocárdio. Outros efeitos colaterais raros também foram relatados, incluindo miocardite, pericardite, arritmias incluindo fibrilação atrial, prolongamento do intervalo QT, insuficiência cardíaca e morte. Essas condições têm sido diretamente associadas ao vasoespasmo devido à disfunção endotelial.^1^, ^2^

Este relato de caso detalha o diagnóstico e manejo da cardiotoxicidade do 5-FU após o desenvolvimento de FV em paciente tratado com 5-FU.

### Caso

**Figura 1:**
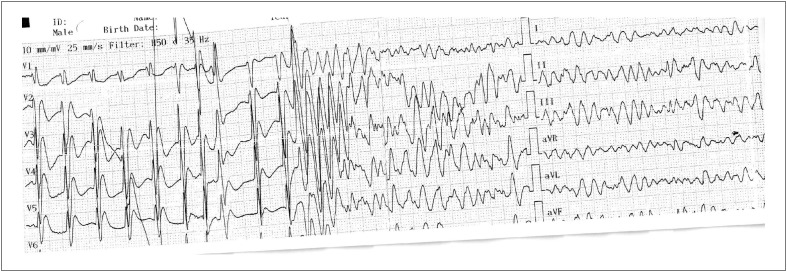
A eletrocardiografia realizada no momento da admissão mostra que houve desenvolvimento de fibrilação ventricular.

Um homem de 65 anos sem histórico de nenhuma doença crônica além de câncer de língua foi ao pronto-socorro com contrações insensíveis estando em casa. O paciente estava na 40ª hora de terapia infusional com bomba de fluorouracil no momento da admissão no pronto-socorro, que estava agendada para 46 horas. O paciente nos consultou após ter sido desfibrilado e intubado devido ao desenvolvimento de FV (Figura 1) durante acompanhamento no pronto-socorro. Durante sua avaliação inicial no pronto-socorro, foram notadas alterações inespecíficas em seu eletrocardiograma (ECG) após a desfibrilação.

**Figura 2:**
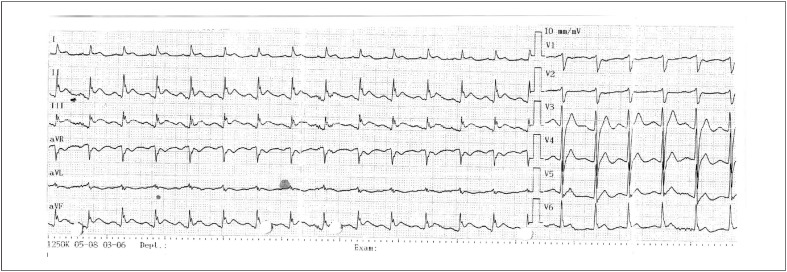
Suspeita de elevação do segmento ST nas derivações inferiores é observada no eletrocardiograma realizado após a desfibrilação.

Os exames laboratoriais revelaram que os valores do hemograma e da bioquímica, incluindo eletrólitos, estavam normais. A FEVE foi de 70% na ecocardiografia à beira do leito e nenhuma patologia valvar adicional foi detectada. O paciente foi admitido em nossa clínica para angiografia coronariana porque o acompanhamento eletrocardiográfico seriado revelou suspeita de supradesnivelamento do segmento ST nas derivações inferiores (Figura 2) e o nível de troponina I aumentou para 144,77 ng/L.

**Figura 3:**
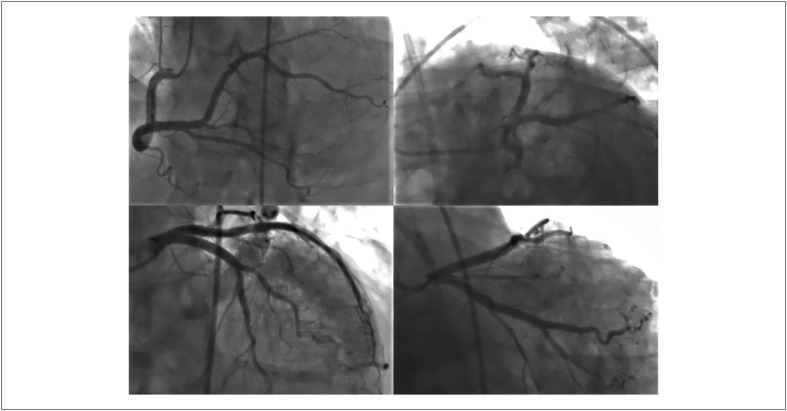
A angiografia coronária não mostra lesão crítica em todas as três artérias coronárias epicárdicas.

A cineangiocoronariografia não revelou estenose crítica (Figura 3).

A terapia de infusão de 5-FU foi interrompida porque se pensava que estava associada à arritmia. Como a fibrilação atrial com resposta ventricular rápida se desenvolveu no período inicial após a angiografia, a infusão de amiodarona e metoprolol oral foram adicionadas ao tratamento e o ritmo sinusal foi alcançado. O paciente foi extubado no segundo dia de acompanhamento em terapia intensiva.

No exame ecocardiográfico detalhado, a FEVE era normal e nenhuma patologia importante foi observada nas válvulas ou câmaras cardíacas. Não foi observado aumento do gradiente na via de saída do ventrículo esquerdo (VSVE) do paciente, cuja espessura do septo interventricular era de 15 mm.

A RMC foi realizada com base nos achados de hipertrofia na ecocardiografia. A RMC mostrou hipertrofia concêntrica do ventrículo esquerdo e não foi detectada obstrução da VSVE. Não foram observados defeitos de perfusão, fibrose ou cicatrizes sugestivas de envolvimento cardíaco.

No acompanhamento clínico do paciente, a FV não apresentou recidiva. Após 15 dias de internação, o paciente recebeu alta hospitalar com amiodarona 200 mg, metoprolol 50 mg e apixabana 5 mg duas vezes ao dia. Acreditava-se que a arritmia do paciente se devia principalmente à cardiotoxicidade induzida pelo 5-FU. Portanto, o paciente não estava agendado para colocação de cardiodesfibrilador implantável (CDI). Nosso paciente não apresentou novo evento arrítmico até o 2º ano de seguimento após a alta. Como isso não foi possível em nosso hospital, não pudemos aplicar um gravador de loop implantável, mas nenhum evento arrítmico foi observado nos exames ambulatoriais de ECG Holter de 24 horas.

## Discussão

Estudos que investigam o vasoespasmo e a cardiomiopatia induzidos pelo 5-FU demonstraram que os níveis de endotelina-1 aumentam e a proteína quinase C é ativada após a exposição ao 5-FU, e foi relatado que isso pode ser responsável pela vasoconstrição.^3^-^5^ Foi sugerido que pode causar isquemia miocárdica ao interferir nos mecanismos de transporte de oxigênio.^6^ Também foi demonstrado que a alfa-fluoro-beta-alanina (FBAL), responsável pela degradação do 5-FU, tem efeito tóxico direto no miocárdio.^7^

Os fatores de risco definidos para o desenvolvimento de cardiotoxicidade incluem irradiação repetida da parede torácica, quimioterapia com múltiplos agentes e doença cardíaca conhecida. Por outro lado, hipertensão, diabetes, hiperlipidemia e tabagismo, fatores de risco para doença coronariana, não foram diretamente associados à cardiotoxicidade.^8^

Nas Diretrizes ESC 2022 para o manejo de pacientes com arritmias ventriculares e prevenção de morte súbita cardíaca, a indicação do CDI para sobreviventes de parada cardíaca por FV definida sem causa reversível é classificada como classe Ia.^9^

Os médicos hesitam em implantar um CDI em pacientes que apresentam morte súbita cardíaca porque consideram o risco de recorrência da morte súbita cardíaca após a alta, mesmo que haja uma causa secundária clara, e para evitar responsabilidade legal. Alguns médicos consideram a implantação do CDI mais segura para o paciente e para a lei e removem o CDI se a arritmia não voltar a ocorrer ou se a FE voltar ao normal. Embora este método pareça ser uma opção tanto para pacientes como para médicos, especialmente em pacientes jovens e pacientes com FEVE limítrofe, é médica e eticamente controverso. Os desfibriladores externos parecem ser a melhor solução para esses pacientes. Como o acesso a esses dispositivos é insuficiente em nosso país, a hospitalização prolongada e a pós-alta sem CDI podem ser uma abordagem lógica para pacientes com FEVE baixa ou FEVE limítrofe normal.

Os desfibriladores cardíacos externos vestíveis ainda não são amplamente utilizados em nosso país; isso parece ser uma desvantagem. Parece ser um aplicativo que permite aos médicos darem alta com segurança a esses pacientes sem CDI. O fato de as causas secundárias terem sido proeminentes em nosso caso, de nenhuma patologia cardíaca evidente que pudesse causar arritmia ter sido encontrada durante o exame detalhado e de a arritmia não ter recidivado durante a internação, que durou até 15 dias, nos levou a acreditar que esse paciente poderia receber alta sem CDI.

Casos de toxicidade por 5 FU foram relatados na literatura, incluindo fibrilação atrial, taquicardia ventricular, FV, insuficiência cardíaca, miocardite, pericardite, cardiomiopatia, síncope e choque cardiogênico.^10^ A utilidade dos bloqueadores dos canais de cálcio e dos tratamentos com nitrato para prevenir o vasoespasmo, a etiologia mais provável entre as modalidades de tratamento para a cardiotoxicidade do 5 FU, é controversa.^11^-^14^ O triacetato de uridina é um pró-fármaco oralmente ativo que é um inibidor competitivo dos metabólitos 5-FU. Foi aprovado pelo FDA em 2015 como antídoto para a toxicidade do 5-FU. No entanto, não há ensaio randomizado sobre seu uso em cardiotoxicidade.^15^-^17^

Outra questão relacionada com a cardiotoxicidade do 5-FU é que reiniciar o tratamento com 5-FU após a cardiotoxicidade coloca os médicos num dilema, pois pode ter consequências mortais. No entanto, as estratégias descritas na literatura para redução da dose ou mudança da terapia de infusão para bolus devem ser consideradas para pacientes que precisam ser reiniciados com 5-FU.^5^, ^18^ Para esses pacientes, a pré e pós-medicação com bloqueadores dos canais de cálcio e nitratos pode prevenir o vasoespasmo coronariano.^14^, ^18^ O monitoramento rigoroso durante a hospitalização inicial, seguido por gravadores de loop implantáveis, pode ser incluído na estratégia para monitorar possíveis eventos arrítmicos.^18^

Embora não exista um consenso comum relativamente ao tratamento da cardiotoxicidade do 5-FU, a abordagem mais adequada pode ser prevenir o desenvolvimento de toxicidade e prevê-la sem consequências graves. Embora não exista uma recomendação clara para o rastreio de rotina, a medição dos níveis de diidropirimidina desidrogenase (DPD) (que aumentam o risco de toxicidade na sua deficiência devido ao seu papel como enzima limitante da taxa no seu catabolismo) pode ser avaliada em pacientes apropriados.^19^ Além do monitoramento da fração de ejeção realizado na prática clínica de rotina, as medições do BNP, do *Strain* Longitudinal Global (GLS) e do Índice Tei podem ser úteis no reconhecimento da cardiotoxicidade subclínica.^20^

## Conclusão

No caso que apresentamos, o paciente desenvolveu parada por FV sem patologia cardíaca identificável que pudesse causar arritmias em todas as investigações etiológicas. A quimioterapia que ele recebeu continha o cardiotóxico 5-FU, conhecido por causar eventos arrítmicos e vasoespasmo. Como o evento ocorreu na 40ª hora da infusão de 5-FU, imaginou-se que o caso fosse FV por cardiotoxicidade do 5-fluorouracil.

Como o evento arrítmico foi reversível, o paciente não recebeu um CDI implantado de acordo com as diretrizes de tratamento do dispositivo. Exames ambulatoriais de ECG Holter não observaram nenhum novo evento arrítmico 24 horas após a alta.

Os efeitos adversos a longo prazo, o manejo de casos e as modalidades de tratamento em sobreviventes de cardiotoxicidade induzida por 5 FU não estão bem definidos. Apresentamos nosso caso sobre esse tema para contribuir com a literatura.
