# Supplementary material for: Uma Causa Rara e Tratamento da Fibrilação Ventricular: Toxicidade do 5-Fluorouracil
Source: Arq Bras Cardiol. 2023 Dec 19;120(12):e20230217. [Article in Portuguese] doi: 10.36660/abc.20230217 (PMC10735207; doi:10.36660/abc.20230217)
Supplement: Translation

# Translation: A Rare Cause and Management of Ventricular Fibrillation: 5-Fluorouracil Toxicity

**Authors:** Kutluhan Eren Hazir, Cenk Sari

**Affiliations:** 1 Konak İzmir Turkey Izmir Provincial Health Directorate Izmir University of Health Sciences Tepecik Training and Research Hospital Ringgold standard institution - Department of Cardiology, Konak, İzmir – Turkey

**Keywords:** Fluorouracil, Ventricular Fibrillation, Medical Oncology

## Introduction

5-Fluorouracil (5-FU) is a pyrimidine antimetabolite used to treat solid tumors.^1^ 5-FU can cause several cardiovascular side effects with a frequency of 1.2-18%.^1^, ^2^ Among these side effects, angina and myocardial infarction are common. Other rare side effects have also been reported, including myocarditis, pericarditis, arrhythmias including atrial fibrillation, QT prolongation, heart failure, and death. These conditions have been directly associated with vasospasm due to endothelial dysfunction.^1^, ^2^

This case report details the diagnosis and management of 5-FU cardiotoxicity upon the development of VF in a patient treated with 5-FU.

## Case

A 65-year-old man with a history of no chronic disease other than tongue cancer applied to the emergency department with senseless contractions at home. The patient was in the 40th hour of fluorouracil pump infusion therapy at the time of admission to the emergency department, which was scheduled for 46 hours. The patient consulted with us after he had been defibrillated and intubated because of the development of VF (Figure 1) during monitoring in the emergency department. During his initial evaluation in the emergency department, nonspecific changes were noted in his electrocardiogram (ECG) after defibrillation.

**Figure 1:** Electrocardiography performed at the time of admission shows that ventricular fibrillation has developed.

Laboratory testing revealed that hemogram and biochemistry values, including electrolytes, were normal. LVEF was 70% on the bedside echocardiography, and no additional valve pathology was detected. The patient was admitted to our clinic for coronary angiography because serial ECG follow-up revealed a suspicious ST-segment elevation in the inferior leads (Figure 2), and the Troponin I level increased to 144.77 ng/L. Coronary angiography did not reveal critical stenosis (Figure 3).

**Figure 2:** Suspected ST-segment elevation in the inferior leads is observed in the electrocardiography performed after defibrillation.

**Figure 3:** Coronary angiography shows no critical lesion in all 3 epicardial coronary arteries.

5-FU infusion therapy was stopped because it was thought to be associated with arrhythmia. Because atrial fibrillation with rapid ventricular response developed in the early period after angiography, amiodarone infusion, and peroral metoprolol were added to the treatment, and sinus rhythm was achieved. The patient was extubated on the second day of intensive care follow-up.

On detailed echocardiographic examination, LVEF was normal, and no major pathology was noted in the valves or cardiac chambers. No gradient increase was noted in the patient's left ventricular outflow tract (LVOT), whose interventricular septal thickness was 15 mm.

Cardiac Magnetic Resonance Imaging (CMRI) was performed based on the hypertrophy findings on echocardiography. CMRI showed concentric hypertrophy of the left ventricle, and no LVOT obstruction was detected. No perfusion defects, fibrosis, or scarring suggestive of cardiac involvement were observed.

At the patient's clinical follow-up, VF did not recur. After 15 days of hospitalization, the patient was discharged with amiodarone 200mg, metoprolol 50mg, and apixaban 5mg twice daily. The patient's arrhythmia was thought to be primarily due to cardiotoxicity induced by 5- FU. Therefore, the patient was not scheduled for implantable cardioverter-defibrillator (ICD) implantation. Our patient had no new arrhythmic event until the 2^nd^ year follow-up after discharge. Since it was not possible in our hospital, we could not apply an implantable loop recorder, but no arrhythmic event was noted in 24-hour ambulatory Holter ECG scans.

## Discussion

Studies investigating 5 FU-induced vasospasm and cardiomyopathy have shown that endothelin-1 levels increase and protein kinase C is activated after 5- FU exposure, and it has been reported that this may be responsible for vasoconstriction.^3^-^5^ It has been suggested that it may cause myocardial ischemia by interfering with oxygen transport mechanisms.^6^ It has also been shown that alpha-fluoro-beta-alanine (FBAL), which is responsible for the degradation of 5 FU, has a direct toxic effect on the myocardium.^7^

Defined risk factors for the development of cardiotoxicity include repeated chest wall irradiation, chemotherapy with multiple agents, and known cardiac disease. On the other hand, hypertension, diabetes, hyperlipidemia, and smoking, which are risk factors for coronary heart disease, have not been directly associated with cardiotoxicity.^8^

In the 2022 ESC Guidelines for the management of patients with ventricular arrhythmias and the prevention of sudden cardiac death, ICD indication for survivors of VF cardiac arrest defined without a reversible cause is classified as class Ia.^9^

Physicians are hesitant to implant an ICD in patients presenting with sudden cardiac death because they consider the risk of recurrence of sudden cardiac death after discharge, even if there is a clear secondary cause, and in order to avoid legal liability. Some physicians consider ICD implantation safer for the patient and the law and remove the ICD if the arrhythmia does not recur or if the EF returns to normal. Although this method seems to be an option for both patients and physicians, especially in young patients and patients with borderline LVEF, it is medically and ethically controversial. Wearable defibrillators seem to be the best solution for these patients. Because access to these devices is insufficient in our country, prolonged hospitalization and post-discharge without an ICD may be a logical approach for patients with low LVEF or borderline normal LVEF.

Wearable external cardiac defibrillators are not yet widely used in our country; this seems to be a disadvantage. It seems to be an application allowing physicians to safely discharge these patients without an ICD. The fact that secondary causes were prominent in our case, no obvious cardiac pathology that could cause an arrhythmia was found during the detailed examination, and the arrhythmia did not recur during the hospitalization, which lasted up to 15 days, led us to believe that this patient could be discharged without an ICD.

Cases of 5 FU toxicity have been reported in the literature, including atrial fibrillation, ventricular tachycardia, VF, heart failure, myocarditis, pericarditis, cardiomyopathy, syncope, and cardiogenic shock.^10^ The utility of calcium channel blockers and nitrate treatments to prevent vasospasm, the most likely etiology among the treatment modalities for 5 FU cardiotoxicity, is controversial.^11^-^14^ Uridine triacetate is an orally active prodrug that is a competitive inhibitor of 5- FU metabolites. It was approved by the FDA in 2015 as an antidote for 5-FU toxicity. However, there is no randomized trial of its use in cardiotoxicity.^15^-^17^

Another issue related to 5-FU cardiotoxicity is that re-initiating 5-FU treatment after cardiotoxicity puts physicians in a dilemma as it may have mortal consequences. However, the strategies described in the literature for dose reduction or switching from infusion to bolus therapy should be considered for patients who need to be restarted with 5-FU.^5^, ^18^ For these patients, pre- and post-medication with calcium channel blockers and nitrates can prevent coronary vasospasm.^14^, ^18^ Close monitoring during initial hospitalization, followed by implantable loop recorders, can be included in the strategy to monitor possible arrhythmic events.^18^

Although there is no common consensus regarding the management of 5-FU cardiotoxicity, the most appropriate approach may be to prevent the development of toxicity and to predict it without serious consequences. While there is no clear recommendation for routine screening, measurement of Dihydropyrimidine dehydrogenase (DPD) levels (which increase the risk of toxicity in its deficiency due to its role as a rate-limiting enzyme in its catabolism) can be evaluated in appropriate patients.^19^ Besides ejection fraction monitoring performed in routine clinical practice, BNP, Global Longitudinal Strain (GLS), and Tei Index measurements may be useful in recognizing subclinical cardiotoxicity.^20^

## Conclusion

In the case we presented, the patient developed VF arrest without identifiable cardiac pathology that could cause arrhythmias in all etiologic investigations. The chemotherapy he received contained the cardiotoxic 5 FU, which is known to cause arrhythmic events and vasospasm. Since the event occurred in the 40^th^ hour of the 5-FU infusion, it was thought that the case was VF due to 5-Fluorouracil cardiotoxicity.

Because the arrhythmic event was reversible, the patient was not implanted with an ICD according to the device treatment guideline. Ambulatory Holter ECG scans observed no new arrhythmic event 24 hours after discharge.

Long-term adverse effects, case management, and treatment modalities in survivors of 5 FU-induced cardiotoxicity are not well defined. We presented our case on this topic to contribute to the literature.
